# Intrauterine Zn Deficiency Favors Thyrotropin-Releasing Hormone-Increasing Effects on Thyrotropin Serum Levels and Induces Subclinical Hypothyroidism in Weaned Rats

**DOI:** 10.3390/nu9101139

**Published:** 2017-10-18

**Authors:** Viridiana Alcántara-Alonso, Elena Alvarez-Salas, Gilberto Matamoros-Trejo, Patricia de Gortari

**Affiliations:** Molecular Neurophysiology Laboratory, Department of Neurosciences Research, National Institute of Psychiatry Ramon de la Fuente Muñiz, Calzada México-Xochimilco 101, Col. San Lorenzo Huipulco, Mexico City C.P. 14370, Mexico; viridiana15kr@hotmail.com (V.A.-A.); alvareze@imp.edu.mx (E.A.-S.); gilmtrejo@yahoo.com.mx (G.M.-T.)

**Keywords:** Zn deficiency, TRH, TSH, subclinical hypothyroidism

## Abstract

Individuals who consume a diet deficient in zinc (Zn-deficient) develop alterations in hypothalamic-pituitary-thyroid axis function, i.e., a low metabolic rate and cold insensitivity. Although those disturbances are related to primary hypothyroidism, intrauterine or postnatal Zn-deficient adults have an increased thyrotropin (TSH) concentration, but unchanged thyroid hormone (TH) levels and decreased body weight. This does not support the view that the hypothyroidism develops due to a low Zn intake. In addition, intrauterine or postnatal Zn-deficiency in weaned and adult rats reduces the activity of pyroglutamyl aminopeptidase II (PPII) in the medial-basal hypothalamus (MBH). PPII is an enzyme that degrades thyrotropin-releasing hormone (TRH). This hypothalamic peptide stimulates its receptor in adenohypophysis, thereby increasing TSH release. We analyzed whether earlier low TH is responsible for the high TSH levels reported in adults, or if TRH release is enhanced by Zn deficiency at weaning. Dams were fed a 2 ppm Zn-deficient diet in the period from one week prior to gestation and up to three weeks after delivery. We found a high release of hypothalamic TRH, which along with reduced MBH PPII activity, increased TSH levels in Zn-deficient pups independently of changes in TH concentration. We found that primary hypothyroidism did not develop in intrauterine Zn-deficient weaned rats and we confirmed that metal deficiency enhances TSH levels since early-life, favoring subclinical hypothyroidism development which remains into adulthood.

## 1. Introduction

Zinc (Zn) deficiency is a public health problem due to its increasing prevalence not only in underdeveloped countries but also in first world countries [1,2,3]. Gestating and lactating women are the most affected groups [4], leading to Zn malnutrition in their offspring. This impairs fetal development due to the metal’s involvement in a wide diversity of cellular processes: differentiation, reproduction, metabolism and neurogenesis [5].

Given that Zn is the cofactor of a wide number of enzymes, deficiency of the metal alters their activity with severe consequences in children and adults health [6]. For example, Zn-deficient animals and humans present growth retardation, cold sensitivity and decreased metabolic rate [7], which are alterations associated with primary hypothyroidism (low thyroid hormone (TH) levels). Moreover, Zn deficiency is also related to psychiatric disturbances such as depression, anxiety, schizophrenia, attention deficit hyperactivity disorder and epilepsy [8,9,10,11].

The hypothalamic-pituitary-thyroid (HPT) axis is regulated by a negative feedback loop, in such a way that during primary hypothyroidism the decreased TH serum levels lead to an increased release of thyrotropin (TSH) from the adenohypophysis (AH) and to a high synthesis and release of the hypothalamic peptide thyrotropin-releasing hormone (TRH) into the portal blood in order to activate the HPT axis.

There is controversy about the effects of Zn deficiency on HPT axis. Some authors support the development of subclinical hypothyroidism [12,13], while others describe the occurrence of a primary one [14,15]. A previous study from our laboratory in adult rats subjected to intrauterine or postnatal Zn deficiency, showed an increase in serum TSH levels but unchanged T_3_ or T_4_ concentration [16], which argues against primary hypothyroidism as the main alteration of the HPT axis in Zn deficiency. Furthermore, these animals maintained a low body weight as adults, which is not compatible with low circulating TH levels [15,16].

In order to disentangle this controversy, in a previous study we analyzed the effects of a Zn-deficient diet on the activity of a metalloprotease called pyroglutamyl aminopeptidase II (PPII) present in the AH and the mediobasal hypothalamus (MBH) [17,18,19,20], along with its repercussion in the function of the HPT axis of gestating and lactating rats and in their adult offspring [16]. The high specificity of PPII in degrading the hypothalamic peptide TRH when released from the median eminence into the portal blood, as well as its positive regulation by TH levels, has indicated that the activity of this enzyme is part of the negative feedback control of the HPT axis exerted by low TH levels, that would allow a more effective stimulation of TSH release by TRH [21]. However, there is a TH-independent down-regulation of adenohypophyseal PPII activity in intrauterine and postnatal Zn-deficient adult rats and in the MBH in whole-life malnourished weanling and adult animals [16]. This supports the fact that low enzyme activity by itself may be responsible for a greater stimulation of TSH release and serum concentration [16].

Nevertheless, we still have not ruled out if intrauterine Zn deficiency induces an earlier decrease in T_3_ and T_4_ serum concentration since weaning, which could be reducing PPII activity and thus increasing TSH levels previous to our measurements in ten-week old adults. This will be arguing against a PPII regulation only by Zn, supporting that the high TSH concentration observed in Zn-deficient adults results from a previous primary hypothyroidism.

Thus, we here analyzed adenohypophyseal PPII activity, TSH and TH serum levels, as well as TRH concentration in the median eminence of intrauterine Zn-deficient weanling pups. Our findings supported that PPII activity might be modulated independently of the changes in TH levels since weaning. This is relevant to explain the TSH rise when TH concentration is in normal levels as in the subclinical hypothyroidism induced by Zn deficiency. Moreover, since chronic high TSH serum levels are associated with increased lipolysis, low body weight and growing rate, our results help to solve the contradiction of the low body weight maintained by Zn malnourishment in adult animals even when T_3_ and T_4_ levels do not change.

## 2. Materials and Methods

### 2.1. Animals and Diets

All procedures described in the present study were approved by the Ethics Committee and Project Commission of the INPRFM, which follows the regulations established in the Mexican Official Norm for the use and care of laboratory animals (NOM-062-ZOO-1999).

Ten nulliparous female and six male adult (220–270 g) Wistar rats were obtained from the INPRFM’s animal housing. They were housed in groups (2–3 animals per cage) and allowed to acclimatize to the facilities. They were provided with food (Lab rodent diet #5001, PMI feeds, St. Louis, MO, USA) and tap water ad libitum, and kept in controlled light conditions (lights on from 7:00 to 19:00) and temperature (24 ± 1 °C). After one week of habituation, animals were mated (1–2 females per male) and divided into two groups: control group (C) (*n* = 3 females, 2 males) receiving a diet with 20 ppm of Zn (Purina Mills, LLC/PMI Nutrition International Co., Richmond, IN, USA) and deficient (D) group (*n* = 7 females, 4 males), which received a diet with 2 ppm of Zn (Purina Mills, LLC/PMI Nutrition International Co., Richmond, IN, USA). This Zn content in the diet is known to be sufficient to decreased serum Zn levels after 7 weeks [16]. Except for their Zn content, both diets were the same regarding nutrient composition (19% proteins; 10% lipids; 61% carbohydrates); both groups had ad libitum access to food and distilled water. The mating period lasted 10 days and pregnancy was confirmed by identifying a 10% increase of the initial female body weight (b.w.). Pregnant dams were individually housed throughout gestation and lactation periods and maintained under the same C or D diet. After pregnancy completion, the body weight of the pups was registered at 2, 7, 14 and 21 days of age. At 21 days of age, weaned pups from C (*n* = 6) or D (*n* = 6) dams, were sacrificed by decapitation. Brain and AH were rapidly removed and frozen at −70 °C. Blood was collected and centrifuged at 3000 × *g* for 30 min at 4 °C. Serum was obtained, aliquoted and kept at −70 °C until analysis.

### 2.2. TRH Content in Median Eminence (ME)

Medial basal hypothalamus (MBH) was hand-dissected from frozen brains using Paxinos and Watson Rat Brain Atlas [22]. This region contains the median eminence (ME) (known to be outside the blood brain barrier (BBB)). In order to obtain the MBH, a coronal slice was cut between the coordinates −1.2 to −3.6 mm from bregma, then ME was removed with a scalpel in the ventral part of the slice.

TRH extraction from tissue and the following radioimmunoassay (RIA) were both performed as previously described [23]. TRH content was determined using a previously characterized antibody [24]. The MEs of the pups were homogenized by sonication in 20% acetic acid and centrifuged at 12,000× *g* for 15 min at 4 °C. A 30 μL aliquot of supernatant was kept for protein quantification. The supernatant was obtained and then extracted with 100% methanol and evaporated. Pellets were diluted in RIA buffer (50 mM NaPO_4_ buffer, pH 7.4, containing 0.25% bovine serum albumin (BSA) RIA grade (Sigma-Aldrich, St. Louis, MO, USA), 150 mM NaCl and 0.02% sodium azide (Sigma-Aldrich, St. Louis, MO, USA). Then, a TRH antibody (1:10^6^ dilution) and ^125^I-TRH (5000 cpm) were added. After 36 h of incubation, samples were precipitated with 100% ethanol and centrifuged at 12,000× *g* for 5 min at 4 °C). Supernatant was evaporated in a concentrator (Vacufuge Eppendorf, Brinkmann Instruments, Westbury, NY, USA), and tubes read for 1 min in a radiation counter (LKB Wallace Minigamma Counter, Mount Waverley, Victoria, Australia). A standard curve and an internal standard of hypothalamic extract were included in every assay and parallelism with the curve was verified. Limit of detection was 20 pg, inter and intra-assay variation was 4% and 8%, respectively. Results are expressed in ng TRH/mg protein.

### 2.3. PPII Specific Activity

PPII activity in AH was measured as previously described [16]. Briefly, each AH was homogenized by sonication in 200 μL of 50 mM NaPO_4_ buffer pH 7.5, and centrifuged at 2600× *g* for 15 min at 4 °C. PPII activity was measured in supernatants by using 400 μM pGlu-His-Pro-β-naphtylamine (βNA) (Bachem, Torrance, CA, USA) as substrate, an excess of dipeptidylaminopeptidase IV (EC 3.4.14.15) (Sigma-Aldrich, St. Louis, MO, USA), 0.2 mM *N*-ethylmaleimide (Sigma, St. Louis, MO, USA), an inhibitor of pyroglutamyl peptidase I (EC 3.4.19.3), and 0.2 mM bacitracin (Sigma-Aldrich, St. Louis, MO, USA) an inhibitor of prolyl oligopeptidase (EC 3.4.21.26); in a total volume of 250 μL. Samples were incubated at 37 °C, 50 μL were withdrawn every 60 min and the reaction stopped with 50 μL 100% methanol. Aliquots were diluted to 400 μL with 50% methanol in buffer, before detecting βNA with a fluorometer (Perkin Elmer LS50, Waltham, MA, USA) (excitation 335 nm, emission 410 nm) from a standard curve of βNA (Sigma-Aldrich, St. Louis, MO, USA). A 30 μL aliquot of supernatant was kept for protein quantification. The activity was linear with the time elapsed and referred to supernatant protein content.

### 2.4. Protein Determination

Protein content in AH and ME was determined in 30 μL of homogenate, by digesting with 1 N NaOH for 24 h at room temperature (RT) and protein concentration quantified by folin-phenol reagent method [25].

### 2.5. Serum Hormones’ Determination

TSH determination was performed by RIA using the NIDDK (National Hormone and Pituitary Program) protocol and materials. We used 50 μL of serum in duplicate, samples were diluted 1:3 in RIA buffer (50 mM NaPO_4_, pH 7.5; 150 mM NaCl, 0.25% BSA, 50 mM EDTA), and antibody raised in rabbit against TSH (1:375,000) was added. After 18–24 h incubation at RT, ^125^I-TSH was added (10,000 cpm), followed by 18–24 h incubation at RT. The secondary antibody (goat anti rabbit IgG) was added in 1:40 dilution in PBS (50 mM NaPO_4_, pH 7.5, 150 mM NaCl) plus 2% normal rabbit serum and incubated for 2 h at RT. After adding polyethylene glycol (0.04 g PEG/mL RIA buffer), samples were centrifuged at 5000× *g* for 30 min at 4 °C. Supernatant was aspirated and tubes read for 1 min in a radiation counter (LKB Wallace Minigamma Counter, Mount Waverley, Victoria, Australia). Limit of detection: 5 pg, 13% inter-assay, 6% intra-assay variability.

Five μL of serum were used to determine corticosterone in duplicate (dilution 1:1000) considering the mean as one determination using ICN Biomedicals kit (Aurora, OH, USA). Limits of sensitivity: corticosterone: 20 ng/mL. Intra-assay variability: 7%, inter-assay variability: 8%.

T_4_ and T_3_ were determined in 100 and 25 μL of serum, respectively, using commercial RIA kits (Coat-A-Count Solid-Phase ^125^I RIA. Total T3 DPC TKT31, Total T4 DPC TKT41, Los Angeles, CA, USA) and following manufacturer’s instructions (analytical sensitivity: T_3_, 7 ng/dL and T_4_, 0.25 μg/dL; inter-assay variability: T_3_, T_4_ < 15%, and intra-assay variability T_3_, T_4_ < 9%).

Leptin was determined in order to evaluate if our early-life diet manipulation influences energy balance regulation and satiety. Leptin was measured colorimetrically with a commercially available kit (Merck Rat Leptin ELISA kit, Life Science Merck, Darmstadt, Hesse, Germany using 25 μL of serum diluted 1:4 with an assay buffer and following the manufacturer’s instructions (limit of detection: 4.76 pg/mL; inter-assay variability (8%), intra-assay variability (7%)).

### 2.6. Statistical Analysis

Body weight changes between control and Zn-deficient pups during lactation were analyzed by repeated measures ANOVA. Kolmogorov-Smirnov tests for normality were used for each of the biochemical variables (PPII specific activity in AH, TRH ME content, TSH, corticosterone, T_3_, T_4_ and leptin serum levels) and then Mann-Whitney U tests were performed in order to identify the statistical differences of these variables between groups. A *p* < 0.05 was considered as statistically significant. A variable interdependence between TRH content and leptin serum levels was analyzed given the positive effect of leptin on TRH synthesis and release [26,27]. A correlation coefficient with magnitude ≥0.8 was considered as strong correlation.

## 3. Results

### 3.1. Body Weight

**Figure 1:**
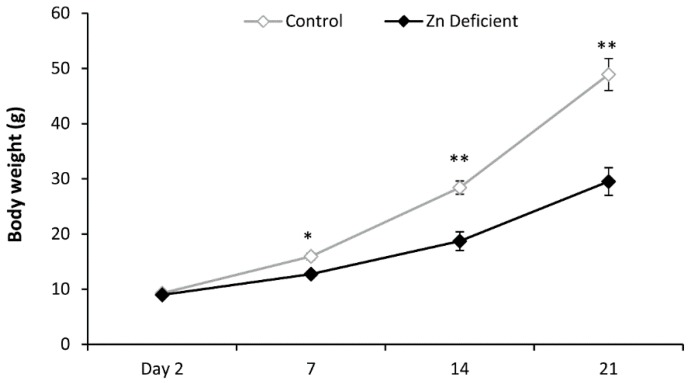
Body weight of control and zinc-deficient (Zn-deficient) pups during lactation period. Values are the mean ± standard error of mean (SEM) of grams of body weight, (*n* = 6/group). Fisher’s post-hoc test showed significant differences: * *p* < 0.01, ** *p* < 0.001 vs. control group.

Body weight at birth was similar between control and Zn-deficient animals; however from post-natal day 7 and until post-natal day 21, the body weight of deficient pups was 30% lower on average, when compared to control offspring (100%). Repeated measures ANOVA showed an effect of treatment (*F*_(1,30)_ = 26.548 *p* < 0.001); time (*F*_(3,30)_ = 219.015 *p* < 0.0001) and interaction between variables (*F*_(3,30)_ = 22.509 *p* < 0.0001) (Figure 1).

### 3.2. Biochemical Determinations

Adenohypophyseal PPII specific activity of Zn-deficient pups showed a trend to decrease, but there was no statistically significant difference between groups (Zn-deficient = 472.5 ± 67 pmol of βNA min/mg of protein vs. controls = 631.2 ± 194 pmol of βNA/min/mg of protein). TRH content of ME in the Zn-malnourished group decreased to 4.7 ± 1.8 ng of TRH/mg protein, when compared to control values: 22.36 ± 6.2 ng of TRH/mg protein. Low accumulation of TRH in the synaptic terminals of hypothalamic paraventricular neurons (median eminence) is associated with a high release of the peptide into the portal blood, mainly because the antibody used is able to detect changes in TRH concentration only in the intracellular compartment.

**Figure 2:**
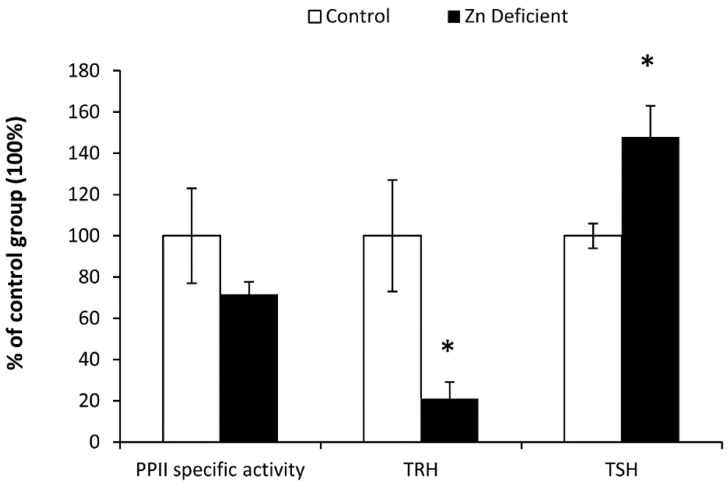
Adenohypophyseal pyroglutamyl aminopeptidase II (PPII) specific activity, median eminence thyrotropin-releasing hormone (TRH) content and thyrotropin (TSH) serum levels of Zn-deficient and control pups at weaning. Control values for PPII specific activity: 631.2 ± 194 pmol βNA/min/mg prot; TRH: 22.36 ± 6.2 ngTRH/mg proteins; TSH: 1.66 ± 0.1 ng/dL. Values are the mean ± SEM of percentage of control values (=100%), (*n* = 6/group).* *p* < 0.01 vs. controls.

Increased TSH serum levels in Zn-deficient pups confirmed the enhanced release of TRH: the Zn-deficient group presented TSH levels of 2.32 ± 0.2 ng/dL, whereas those of the controls were: 1.66 ± 0.1 ng/dL. Mann-Whitney U-test for TRH showed a U = 42 equivalent to a Z = −2.6 with a *p* value < 0.01 and that of TSH was U = 40 equivalent to a Z = −2.714 with a *p* value < 0.01 (Figure 2).

Zn deficiency did not affect T_3_, T_4_ or corticosterone serum levels.

In contrast, circulatory leptin levels decreased to 43.5 ± 7% compared to those of controls (100%); Mann-Whitney U analysis for leptin showed a value for U = 15 that is equivalent to a Z = −2.021 with a *p* value < 0.05 (Table 1). We observed a strong positive linear correlation between median eminence TRH and leptin serum concentration (*n* = 8 rats; correlation coefficient = 0.825; Z = 2.62; *p* < 0.01).

## 4. Discussion

In this study, TH levels of Zn-deficient rats were normal, thus intrauterine metal malnourishment failed to induce primary hypothyroidism since weaning. This was in agreement with the unchanged levels of T_3_ and T_4_ observed in adult rats subjected to Zn deficiency during the prenatal and postnatal periods. Overall, these findings supported the conclusion that high TSH levels and the development of subclinical hypothyroidism in adults [16] and weanling rats, are not a response to low T_3_, but to other factors that we discuss below.

The weight loss at weaning observed in the offspring of dams eating a Zn-deficient diet could be associated to the increase in TSH serum concentration, even when TH did not increase. TSH receptors have been identified in adipocytes and its activation by TSH increases lipid degradation [28,29]. It is well known that high levels of TSH have a down-regulatory effect on its receptors in the thyroid cell [30,31], which may account for the thyroid’s lack of response to the TSH released, thus T_3_ and T_4_ serum concentration did not change in Zn-deficient pups.

Given that body weight of Zn-deficient animals was similar to that of controls at birth, it is likely that dams in the malnourished group compensated Zn availability for the offspring by homeostatic adjustments, at the expense of their own metal content in bone and muscle [32,33] and also, by delivering small litters. The lower body weight in the first week of life seemed also to be a consequence of the known anorexic effect of Zn deficiency [34]. Weanling pups from Zn-deficient dams have a reduction of 40% milk intake on average [35]. We did not evaluate milk consumption since this is a rather stressful procedure, but in our previous study, we describe a decreased food intake in prenatally and postnatally Zn-deficient rats after weaning [16]. This anorexic effect along with the high TSH concentration contributed to the weight loss of malnourished pups.

It was noteworthy that the evident low body weight of Zn-deficient pups was not able to reduce HPT axis activity and to decrease TH levels as has been proposed [36,37]. In contrast, our data supported that hypothalamic neurons are firstly responding to low nutrient availability and as a consequence, metabolic rate is adjusted through HPT axis function modulation. For example, negative energy balance decelerates HPT axis and lipids waste in adults, but in weanling rats such adaptation is not successful [38]. Similarly, in this study, the low Zn intake seemed to activate TRH release from the ME (discussed below) and to increase TSH serum levels, with no abatement of the metabolic rate.

The high TSH serum levels found in Zn-deficient weanling rats was coincident with that previously found in prenatally or postnatally deficient adults [16]. TSH release is known to result from a high release of its secretagogue TRH, or, by a low concentration of TH. In the first case, TRH by activating its receptor (TRH-R1) expressed in thyrotrophs, leads to an enhanced TSH release [39,40]. In the second case, the low concentration of T_3_ favors the detachment of a nuclear thyrotropin receptor (TR) from the TSH gene promoter, in such a way that the expression of the hormone is uninhibited [41].

In this study the effect of low TH concentration appears not to be responsible for the increase in TSH serum levels of Zn-deficient animals. This result argues against the proposed primary hypothyroidism and slow growth induced by low Zn dietary content [15] and, against the decreased Zn concentrations in individuals with primary hypothyroidism [42].

Therefore, a more plausible explanation for the increased circulation of TSH was that the ME nerve terminals of Zn-deficient pups maintained a high TRH concentration in the portal blood. Indeed, pups showed low TRH levels in the tissue containing the nerve terminals from the hypothalamic paraventricular nucleus (PVN) cells. That can be interpreted as a high release given that the titer of the antibody used allows for the quantification of the peptide concentration only in the intracellular compartment, which is greater than that of the extracellular one [43]. When TRH levels decay in the ME along with a high concentration of TSH in serum, then an increased release of the peptide can be assumed.

Trying to identify which factors might be involved in the higher release of hypophysiotropic TRH, we analyzed leptin and corticosterone serum levels, which respectively decrease and increase in a negative energy balance. Both are modulators of TRH synthesis in the hypothalamus [26]. Indeed we found decreased leptin serum levels in Zn-deficient animals, which should be decreasing the function of HPT axis as an advantageous adaptation to their low body weight and reduced energy reservoirs. In contrast and as previously mentioned, a high release of TRH was observed along with increased TSH serum levels that led to further energy utilization; thus we assumed that leptin signaling was impaired in Zn-deficient group.

The shift between leptin/corticosterone levels during fasting elevates the activity of type-2 deiodinase, an enzyme residing in the ependymal cells of the third ventricle that is able to increase T_3_ hypothalamic concentration, which in turn down-regulates TRH expression and release [44], and also would decrease TSH serum levels. However, a Zn-deficient diet did not increase circulating corticosterone in pups. Thus, it is unlikely that alterations found in HPT axis were stress mediated. It is possible that an unchanged type-2 deiodinase activity was not able to reduce TRH expression, avoiding the inhibitory effect of this enzyme on the metabolic rate. Association between high TRH release with activation of its mRNA expression is assumed after observing the coordination of those processes in PVN TRHergic neurons by stimuli, such as suction in lactating rats [45], cold [45,46] and dehydration-induced anorexia [47].

TRH release is modulated by glutamate, of which neurotransmission is altered by brain Zn availability. Zn is able to directly and specifically inhibit responses of glutamate by altering the NMDA receptors [48,49,50]. Thus, low Zn levels might be activating the glutamate receptors expressed in TRHergic neurons [51] and stimulating the release of peptides, inducing different behavioral outcomes [52,53], although this deserves further study.

The other direct effect that Zn deficiency could have to induce the greater actions of TRH on the AH of weaned offspring was the down-regulation of PPII specific activity (the TRH-degrading enzyme) that we have already observed in the MBH of malnourished pups [16]. Low PPII activity most likely decreased TRH degradation and contributed to the greater TRH effect on AH and on TSH release of Zn-deficient pups by enhancing the peptide content in the portal blood and its access to thyrotrophs.

Even when it is known that TH exerts a positive regulation of PPII, its decreased activity in the MBH could not be attributed to T_3_ or T_4_ given that these hormones did not change, but instead it can be attributed to the low Zn availability. This is supported by the Zn dependence of PPII activity that has already proven to be modulated by dietary Zn in adults, as happens for other enzymes, such as alcohol dehydrogenase of the liver [54], alkaline phosphatase [55] and angiotensin-converting enzyme [56,57,58].

In contrast to what we observed in the MBH (in pups and adults in our previous study) and in AH (adults) [16], we did not find a down-regulation of PPII activity in the AH of Zn-deficient weanling pups, which supports a tissue and age specific effect of metal availability on enzyme function. The ontogeny of hypothalamic PPII activity reveals a maximum functionality at postnatal day 8 [59]. By this time, the BBB had already developed in a Zn-deficient environment, avoiding Zn entry through its transporters present in this barrier [60] and affecting PPII activity in the MBH [16]. Moreover, PPII is more active in the hypothalamus when compared to the AH [59], therefore a higher supply of Zn may be required in this brain region in order to assure enzyme activity. On the other hand, Zn-deficient dams might still be able to provide sufficient Zn for PPII activity to function in the AH.

## 5. Conclusions

We conclude that the subclinical hypothyroidism associated with Zn deficiency in adults [12,13,61] is developed at least after weaning; that this is not due to an early decrease in TH serum levels at weaning; and thus, that the increased TSH concentration is not a response to a primary hypothyroidism.

Our data better support the conclusion that Zn deficiency has a direct effect on TRH release, along with a decreased degrading activity of MBH PPII since weaning and later in the AH, which favored TRH stimulation of its receptor in the thyrotrophs inducing a high release of TSH. These elevated TSH levels may be responsible for the low body weight of Zn-deficient pups and may have long lasting effects on animals’ health. For example, high TSH is a possible indicator of Zn deficiency and subclinical hypothyroidism that have been associated to a risk of developing overt hypothyroidism [62,63]. Additionally, high circulating TSH levels are related to several comorbidities such as metabolic syndrome, being overweight, insulin resistance, cardiovascular risk, and dyslipidemia, amongst others [64,65,66,67,68,69].

## Figures and Tables

**Table 1:** Hormones serum concentrations of Zn-deficient and control pups at weaning.

Group	T_3_ (ng/dL)	T_4_ (μg/dL)	Leptin (ng/mL)	Corticosterone (ng/mL)
Control	8.17 ± 0.15	8.5 ± 1	4.3 ± 0.8	149 ± 19
Zn-deficient	7.35 ± 0.49	8.86 ± 1.1	1.86 ± 0.2 *	99 ± 17

Values are the mean ± SEM (*n* = 4–6/group); * *p* < 0.05 vs. control group.
